# Endoscopic endonasal approach for a tuberculum sellae meningioma with optic canal involvement

**DOI:** 10.3171/2025.10.FOCVID25151

**Published:** 2026-01-01

**Authors:** Shunsuke Shibao, Yusuke Morinaga, Takeshi Hongo, Sotaro Oshida, Takashi Kashiwagi, Yasuhiro Tsunemi, Ryu Kurokawa, Tsuguhisa Nakayama, Hiroyoshi Akutsu

**Affiliations:** Departments of 1 Neurosurgery and; 2 Otorhinolaryngology-Head and Neck Surgery, Dokkyo Medical University, Tochigi, Japan

**Keywords:** tuberculum sellae meningioma, endoscopic endonasal approach, optic canal decompression, visual recovery, skull base surgery

## Abstract

The endoscopic endonasal approach has emerged as an effective and minimally invasive technique for the management of tuberculum sellae meningiomas. A 53-year-old woman with progressive visual deterioration due to a tuberculum sellae meningioma extending into the optic canal underwent endoscopic endonasal resection with optic canal decompression. Gross-total removal was achieved using a "French-door" dural opening and angled dissection, resulting in Simpson grade II resection with the preservation of neurovascular structures. Optic canal opening enabled safe tumor removal and significant visual improvement. Reconstruction employed sutured fascia lata grafts and a sphenoid sinus mucosal flap resulted in no postoperative cerebrospinal fluid leakage.

The video can be found here: https://stream.cadmore.media/r10.3171/2025.10.FOCVID25151

## Transcript

### 0:25 Clinical Presentation.

The patient was a 53-year-old woman with a history of bilateral angle-closure glaucoma. Three months before surgery, she developed left visual field deficit and decreased visual acuity (20/200). MRI demonstrated a tuberculum sellae lesion. Endocrine evaluation revealed normal pituitary function.

### 0:47 Preoperative Visual Field.

Preoperative visual field testing demonstrated temporal hemianopia in the left eye.

### 0:53 Preoperative MRI.

Gadolinium-enhanced MRI revealed a tuberculum sellae meningioma with predominant left-sided lateral extension and suspected invasion of the left optic canal. On sagittal images, the tumor was characterized by low-lying extension from the tuberculum sellae into the sella.

### 1:11 Indication and Approach Selection.

Because the patient exhibited progressive symptoms, surgical resection was indicated with the objective of achieving optic nerve decompression and gross-total tumor removal.^1,2^ Given the low-lying extension of the tumor from the tuberculum sellae into the sella, the endoscopic endonasal approach was considered more advantageous than a transcranial route and was therefore selected. Gross-total resection was pursued through anterior skull base dural opening, including optic canal decompression.

### 1:43 Positioning and Preparation.

The patient was positioned supine with the upper body elevated 15°, the head slightly rotated to the right and tilted to the left, and secured in a 3-point Mayfield head holder. Neuronavigation guidance and visual evoked potential monitoring were employed throughout the procedure.

### 2:00 Otolaryngological Phase.

The otolaryngology team performs a Kilian incision on the right septal mucosa, followed by subperiosteal dissection. The bony nasal septum is removed, providing access to the anterior wall of the sphenoid sinus, which is partially resected. The natural ostium of the sphenoid sinus is identified, and a rescue flap incision extending to the anterior margin of the left middle turbinate is created using electrocautery. The bilateral posterior ethmoid sinus is opened and unified with the sphenoid sinus via the left middle meatus and the right superior meatus. The palatovaginal artery and vein are coagulated and divided, and the bilateral palatovaginal canals are exposed.^3^ The olfactory mucosa of the septum is subperiosteally dissected off while preserving the olfactory filaments, and the upper portion of the anterior wall of the sphenoid sinus is removed.^4^

### 3:25 Neurosurgical Phase—Sphenoidotomy and Bony Removal.

The sphenoid septum is removed and the cavity is flattened to better visualize the bony prominences within the sphenoid sinus. Eggshell drilling is then performed around the planum sphenoidale, the tuberculum sellae, the sella, the medial opticocarotid recess, and the optic canal. The bony roof of the optic canal is widely removed, which allows safe and reliable dissection of the laterally extending portion of the tumor. Ultimately, a "chef’s hat"–shaped bony window is created.

### 4:12 Dural Opening and Tumor Resection.

A transverse incision is made in the dura over the sella, followed by a vertical extension. At the superior margin of the optic canal, a transverse dural incision is performed. To allow direct visualization of the tumor extension within the optic canal, the optic nerve sheath is incised under angled endoscopic observation, proceeding from medial to lateral to prevent optic nerve injury. To avoid damage to the ophthalmic artery, the incision is confined to the superior half of the optic nerve sheath. This dural opening is then connected to a vertical incision of the anterior skull base dura, which is coagulated and divided at the anterior intercavernous sinus and subsequently joined with the sellar incision. Opening of the diaphragma sellae allows the dura to be unfolded in a "French-door" fashion. The dural flaps are fixed in place with fibrin glue to maintain exposure.

The tumor is dissected from the arachnoid using L-shaped hooks and golf club–shaped curettes, approaching its anterior margin from the left superior side. Because of its relatively soft consistency, internal debulking is performed as needed with an ultrasonic aspirator. The tumor is easily separated from the right optic nerve; however, it extends laterally into the left optic canal. The attachment site is carefully detached from the dura. The tumor is meticulously dissected from the pituitary stalk, optic chiasm, and anterior cerebral arteries while preserving the intervening arachnoid planes. Inferiorly, dissection from the superior hypophyseal artery is performed with particular care, allowing preservation of this vessel which feeds the visual apparatus. Circumferential dissection is completed, and gross-total removal is achieved. Tumor wedged between the left optic nerve and the internal carotid artery and extending into the optic canal is removed under 30° endoscopic visualization. The dural attachment is not resected, and the residual dura is treated with coagulation, resulting in a Simpson grade II resection. Indocyanine green video angiography confirms patency of the anterior cerebral artery, superior hypophyseal artery, and pituitary stalk. Visual evoked potentials show no waveform changes bilaterally before or after resection.

### 7:04 Dural Reconstruction.

An inlay graft of fascia lata harvested from the right thigh is sutured in place, and the extracavitary knotting and tying is performed using a sliding-knot technique.^5^ The French door–opened dural flaps are reinforced with additional sutures. After fixation with fibrin glue, an onlay fascia lata graft is applied, followed by coverage with a sphenoid sinus mucosal flap. The bilateral olfactory mucosae are completely preserved. Finally, a dural sealant is applied to secure the reconstruction, and the procedure is completed. No postoperative prophylactic spinal drainage was employed.

### 7:57 Postoperative Visual Field.

Postoperative visual field testing demonstrated improvement of the left temporal hemianopia. Postoperative visual acuity improved to 20/16 in both eyes.

### 8:08 Postoperative MRI.

Postoperative MRI confirmed gross-total resection of the tumor. Olfaction was preserved postoperatively, and no cerebrospinal fluid leakage or infection was observed. Histopathological examination revealed a meningothelial meningioma, WHO grade I.

### 8:26 Necessity of Dural Attachment Resection in Tuberculum Sellae Meningioma.

Because resecting the dural attachment surrounding the distal dural ring of the internal carotid artery is anatomically difficult, and given the recent report by Wang et al. that there is no significant difference in recurrence rates between Simpson grades I and II resection, the complete dural attachment resection was avoided.^6^

### 8:45 Multilayered Skull Base Reconstruction in High-Flow CSF Leak Cases.

This table summarizes multilayered skull base reconstruction methods for intraoperative high-flow cerebrospinal fluid leak cases.^7–10^ Dural suturing with fat or fascia provides strong watertight closure and stable graft fixation, often reducing the need for a nasoseptal flap or lumbar drainage, though it is technically demanding.^7,8^ Nasoseptal flap–based multilayer reconstruction remains the gold standard for intraoperative high-flow cerebrospinal fluid leak cases, but its drawbacks include possible flap necrosis or displacement and increased nasal morbidity.^9^ Collagen matrix offers the advantage of reducing donor-site morbidity.^10^ In our institution, intraoperative high-flow cerebrospinal fluid leaks are repaired with a sutured inlay fascia lata with French-door dural closure plus nonsutured onlay fascia lata covered with a sphenoid sinus mucosal flap, achieving reliable closure even without using a nasoseptal flap, while the nasoseptal flap is reserved for high-risk cases such as chordoma.^7^ In high-flow cases, the reported postoperative cerebrospinal fluid leak rate is 6.8% with fascial suturing,^7^ compared with 7.1% in nasoseptal flap–based multilayered reconstruction using autograft.^9^

## Acknowledgments

We used ChatGPT to edit part of the English text.

## Disclosures

The authors report no conflict of interest concerning the materials or methods used in this study or the findings specified in this publication.

## Author Contributions

Primary surgeon: Akutsu. Assistant surgeon: Shibao, Morinaga, Hongo, Oshida, Kashiwagi, Tsunemi. Editing and drafting the video and abstract: Akutsu, Shibao, Hongo, Kurokawa. Critically revising the work: Akutsu, Shibao, Kurokawa, Nakayama. Reviewed submitted version of the work: Akutsu, Hongo, Kurokawa, Nakayama. Approved the final version of the work on behalf of all authors: Akutsu. Supervision: Akutsu, Nakayama. Perioperative care: Kurokawa.

## Supplemental Information

### Patient Informed Consent

The necessary patient informed consent was obtained in this study.
